# Tetanus1Read before the Missouri Valley Veterinary Association.

**Published:** 1899-07

**Authors:** L. D. Ryan

**Affiliations:** Emporia, Kansas


### TETANUS.[1]

By L. D. Ryan, V.S.,
EMPORIA, KANSAS.

Causes. It has been demonstrated, after years of study by eminent men in the medical and veterinary profession, that tetanus is decidedly a nervous disease, and is primarily confined to the true spinal system, being produced in many cases by the most trifling injury; or it may follow certain operations, especially where antiseptic precautions were dispensed with. Hence we can readily understand that the specific cause of this disease is the result of some septic material which is very common, especially in soils which are impregnated with excremental matter, and it appears in the form of a thread-like body provided with a head or spore, termed bacillus tetani. This germ gains entrance into the animal economy through wounds, and, as Zuill states, under its influence organic albumin undergoes a series of transformations and generates ptomains which possess properties similar to those of strychnine. Of these alkaloids Brieger has isolated tetanin tetatoxin, spasmotoxin, and toxalbumin.

Forms. Tetanus is divided into traumatic and idiopathic, and into acute and chronic, the first following wounds and other injuries; the second arising without any assignable cause. The first form is generally acute in character, while the latter form is likely to be chronic, and, to the extent of its chronicity, amenable and responsive to treatment. Acute tetanus seldom has a greater duration than four days, the animal perishing either from asphyxia, in a spasm, or otherwise from exhaustion. Chronic tetanus, on the other hand, runs on day after day, most frequently eventuating favorably.

Symptoms and Cause. While this disease affects all animals from the highest to the lowest, I will deal principally with the horse. And in this animal tetanus exhibits its approach in a

[1] Read before the Missouri Valley Veterinary Association.

gradual manner. Attention is first directed to a general malaise, which may be combined with lameness in one of the extremities; then stiffness of the movements of the lower jaw supervenes; mouth becomes dry and sore; mastication and deglutition difficult; membrane nictatans protrudes over eyeball; animal retains standing posture. The masseters, temporals and pterygoid muscles become stiffened and bulging; orbiculars puckered and contracted; eyelids may be closely approximated; spasms of the small nasal muscles lead to dilatation of the nostrils, and that of the upper lip retracts and enlarges the mouth; cervical muscles hard and distended to touch; respiration more or less difficult; animal perspires freely; temperature may be sub-normal or increased before death; pulse, 70–90. Hyperæsthesia and reflex hyper-excitability are observed, which are marked by fear, anxiety, and permanent excitement, and death may finally ensue to relieve the suffering animal.

Treatment. Up to the present date almost every class and description of remedy has been tried for the cure of this disease, without, as yet, any result in the way of a specific, or an approach in such direction, and the only aim of the present practitioner is to alleviate the acute course and make it more chronic, as this adds to the hope of the recovery, thus wearing out, as it were, the force of the active cause. In fact, we know of no remedies that will cut short the progress of a case of tetanus, and therefore the indications are to employ all our efforts to keep our patient alive during the illness through which he is passing. As means to this most desirable end, we should place our patient in comfortable quarters, according to the season of the year, keep him free from excitement, and administer your medicine at the proper intervals. The medicinal agents which are employed in the treatment of this disease are many, including alteratives in the shape of varied preparations of mercury; diuretics, in the form of tincture of cantharides, oleum terbinth, lithia citras, and potas nitras, given in frequent and large doses so as to irritate the urinary passage; sedatives, such as digitalis and hydrocyanic acid, anodynes and narcotics, as opii, morphine, atropine, belladonna, cannabis indica, ether and chloroform internally and by inhalation, in the proportion of 1 to 2, until narcosis commences. Stimulants and antispasmodics, hygienics, and dietetics as support. Calabar bean in sufficient doses to paralyze the voluntary muscles, combined with fluid-extract gelsemium, has been affirmed to be attended with success, and one is impressed with the keen sense of a battle in which nature, properly supported, may reasonably be expected to win.

THE USE OF THE TETANUS ANTITOXIN. This treatment has interested the profession for some time past, and has been most extensively employed by some of our eminent authorities and practitioners, and they report that the mortality has been reduced by the use of this agent in localities where the disease prevails extensively, but as a curative agent it is ineffective. And I will further state that I have found the above medicaments very valuable in the limited cases which have come under my observation, and with their proper use I have met with much success.

